# Comorbidities and characteristics of coronary heart disease patients: their impact on health-related quality of life

**DOI:** 10.1186/s12955-016-0560-1

**Published:** 2016-11-15

**Authors:** Ksenija Tušek-Bunc, Davorina Petek

**Affiliations:** 1 Faculty of Medicine, University of Maribor, Taborska ulica 8, SI 2000 Maribor, Slovenia; 2 Dr. Adolf Drolc Health Centre Maribor, Ul. talcev 9, SI 2000 Maribor, Slovenia; 3 Department of Family Medicine, Faculty of Medicine, University of Ljubljana, Vrazov trg 2, SI 1104 Ljubljana, Slovenia

**Keywords:** Coronary heart disease patient, Health-related quality of life, Vascular comorbidities, Anxiety/depression disorders

## Abstract

**Background:**

Patients with coronary heart disease (CHD) commonly present with more than one comorbid condition, contributing to poorer health-related quality of life (HRQoL). The aim of our study was to identify the associations between HRQoL and patient characteristics, vascular comorbidities and anxiety/depression disorders.

**Methods:**

This observational study was conducted in 36 family medicine practices selected by random stratified sampling from all regions of Slovenia. HRQoL was assessed using the European Quality of Life - 5 Dimensions (EQ-5D) questionnaire and EQ Visual Analogue Scale (EQ-VAS). The associations between HRQoL and patient characteristics stratified by demographics, vascular comorbidities, health services used, their assessment of chronic illness care, and anxiety/depression disorders were identified by ordinal logistic regression and linear regression models.

**Results:**

The final sample included 423 CHD patients with a mean age of 68.0 ± SD 10.8 years; 35.2% were female. Mean EQ-VAS score was 58.6 ± SD 19.9 (median: 60 with interquartile range of 45–75), and mean EQ-5D index was 0.60 ± SD 0.19 (median: 0.56 with interquartile range of 0.41–0.76). The statistically significant predictors of a lower EQ-VAS score were higher family physician visit frequency, heart failure (HF) and anxiety/depression disorders (﻿R² 0.240; F = 17.368﻿﻿; *p* < 0.001). The statistically significant predictor of better HRQoL, according to EQ-5D was higher patient education, whereas higher family physician visit frequency, HF and peripheral artery disease (PAD) were predictors of poorer HRQoL (Nagelkerke *R* ^2^ = 0.298; *χ* ^2^ = 148.151; *p* < 0.001).

**Conclusions:**

Results of our study reveal that comorbid conditions (HF and PAD), family physician visit frequency and years in education are significant predictors of HRQoL in Slovenian CHD patients.

## Background

Coronary heart disease (CHD) is the most common and is, together with stroke, responsible for the largest number of premature cardiovascular diseases (CVDs) deaths. Although this number has been in decline for the last two decades, it has increased in low- and middle-income countries [1–4]. Moreover, patients with CVDs report significantly poorer health-related quality of life (HRQoL) as compared to the population without chronic diseases, similar to patients with other potentially disabling chronic conditions, such as diabetes, arthritis and depression [5, 6].

Poorer HRQoL is associated with different factors, among which are also patient characteristics and comorbid conditions. CHD patients commonly present with more than one comorbid condition (e.g., cerebrovascular disease, peripheral artery disease or heart failure), and often have problems with anxiety and depression [7]. The functional capacity of the patient, quality of life and premature mortality all depend on these comorbid conditions, including the increasing costs of care of primary and comorbid conditions [1, 5]. Vascular comorbidities, heart failure (HF) and anxiety/depression disorders significantly affect not only the outcomes of CHD care but also HRQoL. HRQoL is defined as a person’s health status and is viewed as a continuum of very complex health outcomes where the person is capable of functioning and has a general perception of health and well-being [8]. It is described as (in) compatibility between the actual and desired functional capacity, which is of particular importance in CHD patients [9]. HRQoL is defined by patients’ subjective perception of the effects of the disease (e.g., limited functionality, regular treatment and follow-up) on their everyday life [10, 11]. Besides typical measures, such as management of morbidity and mortality, HRQoL has become an increasingly important outcome measure in the care of elderly patients with chronic conditions. It represents a paradigm shift from survival or cure as a primary endpoint of the patient’s well-being [8] and assessment of the effects of chronic illness on the patient’s everyday life, as well as the final objective of health care efforts in CHD patient population [10, 12, 13]. In addition, HRQoL is also important in predicting long-term prognosis, mortality, and coronary event recurrence [14–16].

HRQoL is measured using generic and disease-specific instruments for three main reasons: to obtain reference or normative population data, to compare the effects of different diseases, conditions and treatments, and to monitor the health of individuals and groups [15–17].

The aim of our study was to identify the associations between CHD patient characteristics, vascular comorbidities, anxiety/depression disorders and HRQoL in CHD patients.

## Methods

### Study design

The study was performed as part of an international study entitled European Practice Assessment of Cardiovascular Risk Management (EPA Cardio) [18, 19], which has a cross-sectional observational design. Its protocol is described in detail elsewhere [18, 19]. In Slovenia, this study was descriptive and exploratory.

### Study sample

A total of 36 family medicine practices across Slovenia participated in this study. Our sample of practices was drawn randomly, using a table of random numbers, and stratified according to the size of practices (small-sized practices with two full-time family physicians or less working on the same location, and large-sized practices with more than two full-time family physicians working on the same location) and urbanisation/practice location (practices located in urban areas with 30,000 inhabitants and more, and those located in rural areas with less than 30,000 inhabitants). In participating practices, the family physicians randomly selected 30 patients with CHD, including those with myocardial infarction, angina pectoris or coronary interventions from the register available at every family medicine practice. At any visit, these patients were given a coded diagnosis (I20-I25) according to the International Statistical Classification of Diseases and Related Health Problems, 10th Revision (ICD-10). Patients with diabetes were excluded from this group to ensure homogeneity of the group. To continue, patients with a terminal disease, cognitive impairment and those who do not understand Slovenian language sufficiently were also excluded from the study. All patients gave written informed consent for participation in the study.

### Instruments

European Quality of Life - 5 dimensions (EQ-5D) [20, 21] was used to obtain the data which represented the main outcomes. EQ-5D is a standardised, generic instrument for describing and valuing health, and it is available in more than 50 languages. This instrument defines health in terms of five dimensions: mobility, self-care, usual activities, pain or discomfort, and anxiety/depression disorders.

Within each dimension, there are three levels: no problems, some problems or extreme problems. The EQ-5D score (indicating HRQoL) ranges from 0 to1 and can be calculated by applying scores from the EQ-5D preference weights elicited from the Slovenian population [22]. The maximum score of 1 indicates the best state of health. Published evidence supports the validity and reliability of the EQ-5D as an outcome measure within CVD [23].

The EQ Visual Analogue Scale (EQ-VAS) was used as a measure of general self-assessment of HRQoL. EQ-VAS is made up of a vertical scale ranging from 1 to 100, where the lowest endpoint is “Worst imaginable health state” and the highest endpoint is “Best imaginable health state”. These two instruments were both part of an extensive patient questionnaire [20, 21].

### Explanatory variables

Using an extended patient questionnaire, which included the EQ-5D, EQ-VAS, MORISKY, PACIC and EUROPEP questionnaires, we obtained data on the following explanatory variables: demographic variables (gender, age, employment, education, marital status), duration of practice involvement, family physician visit frequency per year as well as data on comorbid conditions, which were self-reported, chosen from the list by patients themselves and confirmed by their physicians through an audit of patients’ records, namely angina pectoris, myocardial infarction, stent or coronary by-pass, stroke, and peripheral artery disease. Furthermore, we also obtained data from patients on medication adherence (MORISKY questionnaire) [24], their assessment of chronic illness care (PACIC questionnaire) [25, 26] and their self-rated satisfaction of family practice care and clinical behaviour of their family physician (EUROPEP questionnaire) [27, 28].

Gender, marital status, duration of practice involvement and presence of comorbid cardiovascular diseases were categorical variables. Age (years), education (years), family physician visit frequency medication adherence (sum of points in the questionnaire from 0 to 8), patients’ assessment of chronic illness care (mean value of points from 1 to 5), and patients’ self-rated satisfaction of family practice care (mean value of points from 1 to 5) were continuous variables.

### Power calculation of the study

The available sample size was multiplied by the design effect to account for the cluster research design [29]. For the design effect calculation we used equation 1 + (*m*-1)*ρ given by Donner et al. [30]. The intracluster correlation coefficient (*ρ*) was used from a large sample-study for cluster randomized trials and surveys at the primary care clinic level (a maximum value observed of 0.05) [31]. Considering the mean cluster size (*m*) of 11.8 ± SD 2.1 patients (range 8–15), the design effect was equal to 1.54. The next step included power calculation for the two main statistical methods used in the manuscript. Considering the design effect and using the G*Power software (version 3.1.9) [32], a total of 423 patients was calculated to have a >99% power to detect a significant association for linear regression (using alpha of 0.05, considering 16 predictors and assuming medium effect size of 0.15 [33]). Similarly, considering the design effect, and following Hsieh [34], a total of 423 patients was calculated to have a nearly 90% power to detect a significant association for logistic regression (using alpha of 0.05, medium odds ratio of about 2.5 to 1 [35], and the variance inflation factor of 1.22) . The variance inflation factor, which depends on the squared multiple correlation coefficient (R^2^) relating a specific predictor of interest to the remaining predictors, was calculated according to the instructions in Hsieh et al. [36]. Our study design did not involve one specific predictor of interest, therefore we calculated R^2^ for each predictor in the model and used the maximum value obtained. For continuous predictors selected as dependent variables we applied linear regression to calculate standard R^2^. In case categorical predictors were selected as dependent variables we applied logistic regression for Nagelkerke R^2^.

### Data analysis

We calculated descriptive statistics for patient characteristics. Demographic data were reported as frequencies (%) or mean (SD) values. Only patients with complete data on all explanatory variables were considered in the final model and included in the analyses. The characteristics of these patients were compared with those of patients who were excluded from the analysis using the chi-square test (gender), the independent-samples *t*-test (age) and the EQ-5D score (Mann-Whitney *U* test). Normal distribution of numerical variables was confirmed by the Shapiro-Wilks test.

The main study outcome was HRQoL as measured by two dependent variables of the EQ-VAS and EQ-5D index score, which were also reported as frequencies (%) or mean (SD) and median (interquartile range) values. EQ-5D index score was obtained by applying preference weights from the health value scale defined according to a 3-level EQ-5D scale for Slovenian population [22].

This study tested a total set of 16 potential explanatory variables as predictors of HRQoL: 9 variables representing patient characteristics and 7 variables for comorbidities. Linear regression was used to identify the associations between EQ-VAS, patient characteristics and vascular comorbidities; results were presented as B-coefficient with 95% confidence intervals, standard error and *p*-value. Contrary to the EQ-VAS the EQ-5D scores and its domains were classified as ordinal measurement scales as suggested by Alava et al. [37]. Ordinal logistic regression was used to identify associations between EQ-5D, patient characteristics and vascular comorbidities; results were presented as odds ratio with 95% confidence intervals and *p*-value. The same method was also used for the EQ-5D domains. For the purposes of logistic regression, to reduce the number of ordinal categories, the EQ-5D score was collapsed into 7 levels (1.0, 0.7, 0. 6, 0.5, 0.4, 0.3, 0.2 or lower). Multiple regression modelling included characteristics that were found to be significant by univariate regression only. Statistical analysis was performed using IBM SPSS software, version 21.0 (IBM Corp., Armonk, NY). Statistical significance was set at *p* < 0.05.

## Results

In total, 36 family medicine practices took part in this study, with 768 CHD patients, representing 71.1% of the target sample of 1080 patients. A total of 312 patients refused to participate or did not return questionnaires. To continue, 345 patients were excluded due to unclear coding (*n* = 15), missing data (*n* = 178) or non-fulfilment of inclusion criteria (*n* = 152). As a result, the analysis included 423 patients, which is 55.1% of the sample of eligible patients. Despite the high volume of excluded data, there were no statistically significant differences between the excluded (*n* = 345) and analysed (*n* = 423) samples by mean age (68.7 ± SD 10.5 and 68.0 ± SD 10.8 respectively, *p* = 0.362), gender (64.3% of male patients excluded from the analysis and 64.8% of all male patients participating in the study, respectively, *p* = 0.939), and distribution of patients across practices (*p* = 0.443). The HRQoL of the excluded patients was not significantly different to that of the analysed sample (*p* = 0.329), with mean EQ-5D index scores of 0.60 ± SD 0.19 and 0.59 ± SD 0.18, respectively. Table 1 shows the explanatory variables (socio-demographic patient characteristics and comorbidities) of the final sample of CHD patients.

**Table 1:** Socio-demographic data, patient characteristics and comorbidities

Patient characteristics		*n* = 423	%
Gender	Female	149	35.2
	Male	274	64.8
Employment status^c^	Unemployed	7	1.7
	Other^a^	416	98.3
Marital status	Married	314	74.2
	Other^b^	109	25.8
Years of education^d^		9.7 (SD 2.7)	[4–20]
Family physician visit frequency^d^		6.9 (SD 1.8)	[1–20]
Duration of practice involvement >10 years		313	74.0
Angina pectoris		225	53.2
Myocardial infarction		247	58.4
Stent/coronary bypass		55	13.0
Heart failure		166	39.2
Stroke		32	7.6
Peripheral artery disease		135	31.9
Anxiety/depression		165	39.0
Medication adherence (Morisky)^d^	0 (low)–8 (high)	6.9 (SD 1.2)	[1–8]
Patient satisfaction (EUROPEP)^d^	1 (low)–5 (high)	4.4 (SD 0.6)	[1.7–5.0]
Patient assessment of chronic illness care (PACIC)^d^	1 (low)–5 (high)	3.2 (SD 0.9)	[1.0–5.0]
Age^d^	Female	70.7 (SD 10.4)	[41–94]
	Male	66.5 (SD 10.8)	[43–89]
	Total	68.0 (SD 10.8)	[41–94]

^a^Retired, housewife, employed, self-employed, incapable of work

^b^Divorced, widowed, single

^c^The employment status was omitted from further analysis due to a small number of unemployed CHD patients (low percentage of unemployment)

^d^Continuous variables are presented by mean value, standard deviation and range

Considering the dimensions of the EQ-5D questionnaire, CHD patients indicated that they had the least problems with self-care (mean 1.1 ± SD 0.3), whereas they reported most problems in the Pain/Discomfort dimension (mean 1.8 ± SD 0.5), In addition, CHD patients self-assessed their current health also on a EQ-VAS scale (ranging from 0 to 100; mean 58.6 ± SD 19.9). Median EQ-5D score was 0.56 with interquartile range of 0.41-0.76 and median EQ-VAS score was 60 with interquartile range of 45–75 (Table 2).

**Table 2:** Health-related quality of life score (EQ-5D)

EQ-5D domains	Levels (descriptions) of domains	*n* = 423 (%)	M (SD)
Mobility	No problems	181 (48.2)	1.6 (0.5)
	Some problems	241 (56.9)	
	Confined to bed	1 (0.2)	
Self-care	No problems	380 (89.8)	1.1 (0.3)
	Some problems	40 (90.5)	
	Extreme problems	3 (0.7)	
Usual activities	No problems	170 (40.2)	1.6 (0.6)
	Some problems	234 (55.3)	
	Extreme problems	19 (4.5)	
Pain/discomfort	No pain	101 (23.9)	1.8 (0.5)
	Moderate pain or discomfort	289 (68.3)	
	Extreme pain or discomfort	33 (7.8)	
Anxiety/depression	Not anxious or depressed	258 (61.0)	1.4 (0.5)
	Moderately anxious or depressed	156 (36.9)	
	Extremely anxious or depressed	9 (2.1)	
EQ-5D index score^a^			0.60 (0.19)
EQ-VAS score^b^			58.6 (19.9)

M (SD): mean (standard deviation)

^a^Median value 0.56; interquartile range 0.41–0.76

^b^EQ-VAS score: 100 = Best imaginable health state, 0 = Worst imaginable health state

Table 3 provides details of the association between patient characteristics and EQ-VAS. Each increase by one visit to the family physician per year was associated with a 2.98-point decrease in the EQ-VAS score (B = −2.98, 95% CI = −4.50, −1.46, *p* = 0.002), HF was associated with a 6.27-point decrease in the EQ-VAS score (B = −6.27, 95% CI = −10.13, −2.41, *p* = 0.002) and anxiety/depression disorder was associated with a 10.53-point decrease in the EQ-VAS score (B = −10.53, 95% CI = −14.25, −6.80, *p* < 0.001). Predictors included in the multiple regression model explained 24.0% of the variation in EQ-VAS (R^2^ = 0.240; F = 17.368; *p* < 0.001). Years of education were associated with higher EQ-VAS score and PAD with lower EQ-VAS score by univariate analysis only.

**Table 3:** Multiple linear regression analysis of the predictors of EQ-VAS score

Patient characteristics	Univariate regression^a^			Multiple regression^b^		
	B (95% CI)	SE	*p*	B (95% CI)	SE	*p*
Years of education	4.45 (1.76, 7.15)	1.37	0.001	2.34 (−0.23, 4.91)	1.31	0.074
Marital status	−0.59 (−0.53, -4.14)	2.41	0.805			
Duration of practice involvement >10 years	0.43 (−3.93, 4.79)	2.22	0.846			
Family physician visit frequency	−3.41 (−5.05, -1.77)	0.94	<0.001	−2.98 (−4.50, -1.46)	0.78	0.002
Angina pectoris	−0.77 (−4.55, 3.00)	1.92	0.687			
Myocardial infarction	0.79 (−3.07, 4.64)	1.96	0.689			
Stent/coronary bypass	−2.04 (−7.61, 3.53)	2.83	0.471			
Heart failure	−8.92 (−12.87, -4.96)	2.01	<0.001	−6.27 (−10.13, -2.41)	1.96	0.002
Stroke	0.96 (−6.29, 8.22)	3.69	0.794			
Peripheral artery disease	−5.33 (−9.411, -1.26)	2.07	0.010	−1.76 (−5.69, 2.16)	2.00	0.378
Anxiety/depression	−12.73 (−16.43, -9.03)	1.88	<0.001	−10.53 (−14.25, -6.80)	1.89	<0.001
Medication adherence (Morisky)	0.01 (−11.10, 11.12)	5.65	0.998			
Patient satisfaction (EUROPEP)	2.65 (−0.47, 5.76)	1.58	0.095			
Patient assessment of chronic illness care (PACIC)	1.99 (−0.05, 4.02)	1.03	0.055			

^a^Univariate regression (adjusted for age and gender)

^b^Multiple regression (adjusted for age, gender and for all variables in the model, *R* ^2^ = 0.240; F = 17.368; *p* < 0.001)

Associations between patient characteristics and EQ-5D index are presented in Table 4. For each additional year of education, the odds of a one-level increase in EQ-5D was 1.85 times higher (OR = 1.85, 95% CI = 1.44–2.39, *p* < 0.001). However, for each increase in the number/of visits to the family physician, the odds of a one-level increase in EQ-5D was 0.62 times lower (OR = 0.62, 95% CI = 0.49–0.80, *p* < 0.001). Furthermore for HF and PAD patients, the odds of a one-level increase in EQ-5D was 0.45 times lower (OR = 0.45, 95% CI = 0.31–0.65, *p* < 0.001) and 0.32 times lower (OR = 0.32, 95% CI = 0.22–0.47, *p* < 0.001), respectively. Predictors included in the multiple regression model explained 29.8% of the variation in EQ-5D (Nagelkerke *R* ^2^ = 0.298; *χ* ^2^ = 148.151; *p* < 0.001).

**Table 4:** Ordinal regression analysis of predictors of HRQoL as measured by EQ-5D

Patient characteristics	Univariate regression^a^		Multiple regression^b^	
	OR (95% CI)	*p*	OR (95% CI)	*p*
Years of education	1.94 (1.51–2.49)	<0.001	1.85 (1.44–2.39)	<0.001
Marital status	1.34 (0.88–2.03)	0.174		
Duration of practice involvement >10 years	0.97 (0.66–1.44)	0.895		
Family physician visit frequency	0.56 (0.44–0.72)	<0.001	0.62 (0.49–0.80)	<0.001
Angina pectoris	0.75 (0.54–1.05)	0.096		
Myocardial infarction	0.75 (0.53–1.05)	0.094		
Stent/coronary bypass	0.70 (0.42–1.15)	0.160		
Heart failure	0.35 (0.24–0.50)	<0.001	0.45 (0.31–0.65)	<0.001
Stroke	0.71 (0.37–1.33)	0.278		
Peripheral artery disease	0.29 (0.19–0.42)	<0.001	0.32 (0.22–0.47)	<0.001
Medication adherence (Morisky)	1.85 (0.69–4.96)	0.219		
Patient satisfaction (EUROPEP)	1.27 (0.95–1.68)	0.103		
Patient assessment of chronic illness care (PACIC)	1.03 (0.86–1.23)	0.767		

^a^Univariate regression (adjusted for age and gender)

^b^Multiple regression (adjusted for age, gender and for all variables in the model, Nagelkerke *R* ^2^ = 0.298; *χ* ^2^ = 148.151; *p* < 0.001)

Ordinal regression analysis of predictors of EQ-5D, by individual dimensions of the EQ-5D questionnaire revealed the following results about the predictors for CHD patients:

- Lower mobility (Table 5 in the Appendix) was predicted by higher family physician visit frequency HF, PAD, and anxiety/depression. Higher education of patients was a predictor of better mobility.
- Lower self-care ability (Table 6 in the Appendix) was predicted by higher family physician visit frequency, HF, stroke, PAD, and anxiety/depression. Higher education of patients and marital status were predictors of better self-care ability. Myocardial infarction was a predictor of lower self-care ability in univariate analysis only.
- Lower capacity to perform usual activities (Table 7 in the Appendix) was predicted by higher family physician visit frequency HF, PAD, and anxiety/depression. Higher education of patients was a predictor of better capacity to perform usual activities.
- Being single, having HF, PAD, or anxiety/depression predicted more pain and discomfort (Table 8 in the Appendix), while higher education of patients predicted less pain and discomfort. Higher family physician visit frequency predicted more pain/discomfort in univariate analysis only.
- More anxiety and depression (Table 9 in the Appendix) was predicted by lower patient satisfaction as measured by EUROPEP. Higher family physician visit frequency, stent, HF and PAD were also predictors of more anxiety/depression, while higher education predicted less anxiety and depression.

The analysis of comorbidities as stand-alone predictors of HRQoL (Table 10 in the Appendix) showed HF and anxiety/depression as predictors of lower HRQoL in the EQ-VAS score, while HF and PAD were predictors of lower HRQoL in EQ-5D.

## Discussion

This study investigated the association of HRQoL with CHD patient characteristics and presence of vascular comorbidities and anxiety/depression disorders. We found that the mean HRQoL in this study was somewhat lower than in comparable studies [38–40]. The most consistent predictors were family physician visit frequency, level of education, and the presence of HF, PAD and depression/anxiety as the three most disabling cardiovascular comorbidities.

CHD patients taking part in our study assessed their health state (EQ-5D score = 0.60 ± SD 0.19) similarly to those patients who participated in the international EPA Cardio study (0.7 ± SD 0.2) [38] but somewhat worse as established by the Chinese in their most recent study in this field (overall EQ-5D score = 0.89 ± SD 0.17) [39] and in some other studies (mean EQ-5D score for coronary heart disease patients in the United States was 0.73 ± SE 0.01) and mean EQ-5D score for patients with angina pectoris in the Swedish population in the Stockholm County was 0.70 ± SE 0.02) [40, 41], In addition, in the Chinese and other studies, CHD patients self-assessed their health status on a EQ-VAS scale better compared to our patients (mean EQ-VAS score of 71.6 ± SD 17.7 and 63.4 ± SE 0.6 vs 58.6 ± SD 19.9) [39, 40].

In predicting worse HRQoL on an EQ-VAS scale, we found that statistically significant predictors are: family physician visit frequency, HF, and anxiety/depression disorders. HRQoL prediction according to the EQ-5D questionnaire showed three statistically significant predictors of poorer HRQoL, namely HF, PAD and family physician visit frequency, whereas higher level of education predicted better quality of life.

Different studies showed a negative impact of various conditions (e.g., HF, vascular comorbidities) and socio-demographic factors (including education) on the HRQoL of CHD patients [5, 10, 42], which is comparable to the results of our study. The EPA Cardio study, in which Slovenia also participated, did not explicitly examine the association between individual comorbid conditions and HRQoL but was focused exclusively on the number of different comorbid conditions that had a severe negative impact on HRQoL [38].

In addition to identifying the association between HRQoL and vascular comorbidities in CHD patients, our analysis also included HF, mainly because CHD patients usually survive all complications as a result of better treatment but become more susceptible to the development of HF. The association between HF and HRQoL as measured by EQ-5D and the EQ-VAS score was a statistically significant predictor of poor HRQoL. Moreover, HF was also the outstanding variable in our regression models for all separate domains of EQ-5D, predicting poorer mobility, reduced self-care ability, limited performance of usual activities more pain/discomfort and anxiety-depression disorders. This finding is in line with the findings of several other studies which showed that the quality of life in HF patients is even poorer than in other patients with chronic diseases [43–45].

Besides HF, PAD was another vascular comorbidity that proved to be an important predictor of poorer HRQoL. In addition, in our study PAD was also found to be a predictor of all separate HRQoL domains, namely lower mobility, reduced self-care capacity, limited performance of usual activities, worse general well-being, and anxiety/depression disorders. As early as 30 years ago, Hertzer et al. found that there is high prevalence of CHD among patients with PAD [46]. The quality of life in patients with PAD is typically poorer than in patients with CHD alone [47]. The data obtained in our study regarding PAD were expected, as a CHD patient who also suffers from leg pain and limited mobility in general most definitely has poorer HRQoL, whereas vascular surgery to treat peripheral vascular disease aims at improving HRQoL [48].

In addition to PAD, anxiety/depression disorders also turned out to be a statistically significant independent predictor of poorer quality of life as measured by the EQ-VAS scale which was consistent with the findings of a systematic review of the literature [45]. Anxiety/depression was predictor of poorer quality of life in four domains (mobility, self-care, usual activities and pain/discomfort). Identifying these conditions in CHD patients thus significantly contributes to identifying patients at a greater risk of impaired HRQoL.

Myocardial revascularization procedures (stent placement or coronary artery bypass graft surgery), which reportedly improve HRQoL and general quality of life, are recommended in the guidelines of the American Heart Association (AHA) as one of the primary indications for surgical revascularization [49, 50]. However, despite our expectations, myocardial revascularization procedures did not prove to be a predictor of better HRQoL, but appear only as a statistically significant predictor of better mobility of CHD patients.

Family physician visit frequency was the outlying predictor of both poorer HRQoL (assessed on a EQ-VAS scale) as well as poorer mobility, self-care capacity, limitations in usual activities, more pain/discomfort, and anxiety and depression disorders. We expected that patients, who visit their physician more frequently, would assess their HRQoL as poorer, for this would be in accordance with the findings of other studies [51–54]. Higher family physician visit frequency, which otherwise appears in all dimensions of the EQ-5D questionnaire, as well as in their self-assessment of current health status on a EQ-VAS scale, probably points to unfulfilled needs of CHD patients. In the majority of domains (mobility, usual activities, discomfort and pain) as well as in predicting HRQoL (EQ-5D), higher patient education is statistically significant independent predictor of better HRQoL, which is similar to the findings of the EPA Cardio study [38]. The fact that higher-educated patients demonstrate better HRQoL in our study might be a result of the differences in socio-economic status, which we did not study explicitly here. Socio-economic status appears to be a critical factor that needs to be taken into account in assessing HRQoL [55].

Considering CHD patient characteristics, we, unfortunately, did not find any association between HRQoL and the patients’ assessment of their chronic illness care (PACIC), although other studies have confirmed this association [25].

Higher patient satisfaction with family practice care and clinical behaviour of their family physician (EUROPEP) were predictors of better HRQoL only in one domain of EQ-5D (less anxiety/depression), contrary to the findings of the international EPA Cardio study, which demonstrated a strong positive association between these two variables [38]. Nevertheless, these variables are important, for it highlights the relationship between the physician and the patient, and may improve primary care outcomes [38, 56].

Although medication adherence has an important impact on HRQoL [38, 57, 58], including CHD [59], our study did not show this factor to be a statistically significant predictor of HRQoL.

### Strengths and weaknesses of the study

The weakness of this study was firstly the cross-sectional observational design, which does not assess the impact of changes in demographic and other factors on HRQoL in a specific time period. It also means that there is a risk of reverse causation or, better said, that the cross-sectional study shows associations between the outcome and explanatory variables but not causal associations.

EQ-5D is a simple and widely used questionnaire, but it presents potential problems for statistical analysis due to the fact that distribution is not normal. The distribution of our data was bi-modal, just as described by Alava et al. [37]. Several models have been used in the past to find the best statistical option, with the most frequently used one being a simple linear model. Comparison of the models led to contradictory dilemmas. On this basis we decided to analyse the data using ordinal logistic regression model due to non-linearity of the outcome variable [37].

There are several reasons as to why the sample might not be representative of the Slovenian CHD population: the low response rate, the exclusion of patients who did not understand Slovenian and those with diabetes, family physicians may have failed to include random sample of participants. The final number of patients in the sample was much smaller due to several missing values in the patient questionnaire, which was very long and cognitively demanding in some parts (e.g., PACIC). For the analysis, we used only a part of the instrument. Only patients who completed the questionnaire in full were included in the analysis. We focused on the quality of life in patients with CHD without diabetes, which was an exclusion criterion for the whole EPA Cardio study. However, in our analysis, the exclusion of patients with diabetes represented a limitation in the analysis of HRQoL in CHD patients with multimorbidity. In line with the study’s methodology, we excluded patients who did not understand the Slovenian. These were mostly physical labourers from a low socioeconomic background - a particularly vulnerable group of patients. In addition, a control group without CHD was not included in the study. This means that there is no general non-CHD population sample to compare our sample of CHD patients with, therefore we cannot conclude whether the values for separate EQ-5D dimensions are similar or not to those in the general population without CHD.

Another weakness of the study was that we could not find any correlation with self-assessment of HRQoL and chronic illness care. Both measures are self-reported, therefore a correlation is expected.

The main strength of the study was the random sampling of CHD patients from a computer-generated list available to every family medicine practice. Such patient selection is preferred over the inclusion of sequential visitors to the practice, where both frequent as well as rare visitors have an equal chance of participating in the study. Although, like in many studies, we can not be sure that all participating family physicians followed the inclusion protocol.

Additional strength was our focus on the HRQoL of CHD patients in a representative environment of family medicine practices throughout Slovenia. The obtained data can serve as a basis for further studies.

## Conclusion

HRQoL assessment is an important measure of the quality of patient-oriented care. According to the findings of our study, the most important factors associated with worse HRQoL in CHD patients are comorbidities, primarily signs of advanced CHD manifested by HF and PAD. This means that it is necessary to improve the multidisciplinary approach to the treatment of these patients and to strive to prevent the occurrence and development of these conditions. Furthermore, the impact of anxiety/depression suggests that improved collaborative care between cardiovascular specialists and mental-health care providers is required. The results of this study show that frequent visitors to family medicine practices have poorer HRQoL. These patients should therefore receive more attention and be managed carefully. In addition, the association with years of education and better QoL suggests that there may be socio-economic inequalities involved, which would require further research.

## Appendix

**Table 5:** Ordinal regression analysis of predictors of EQ-5D mobility domain

Patient characteristics	Univariate regression^a^		Multiple regression^b^	
	OR (95% CI)	*p*	OR (95% CI)	*p*
Years of education	1.91 (1.41–2.58)	<0.001	1.80 (1.29–2.51)	0.001
Marital status	1.49 (0.88–2.54)	0.136		
Duration of practice involvement >10 years	0.97 (0.60–1.56)	0.901		
Family physician visit frequency	0.55 (0.40–0.75)	<0.001	0.59 (0.42–0.83)	0.002
Angina pectoris	0.85 (0.56–1.28)	0.439		
Myocardial infarction	0.73 (0.48–1.12)	0.157		
Stent/coronary bypass	1.11 (0.60–2.05)	0.734		
Heart failure	0.34 (0.21–0.53)	<0.001	0.44 (0.27–0.73)	0.001
Stroke	0.71 (0.31–1.60)	0.405		
Peripheral artery disease	0.28 (0.17–0.46)	<0.001	0.33 (0.20–0.57)	<0.001
Anxiety/depression	0.42 (0.27–0.65)	<0.001	0.60 (0.37–0.97)	0.036
Medication adherence (Morisky)	2.97 (0.85–10.32)	0.087		
Patient satisfaction (EUROPEP)	1.32 (0.92–1.89)	0.133		
Patient assessment of chronic illness care (PACIC)	1.07 (0.85–1.34)	0.563		

^a^Univariate regression (adjusted for age and gender)

^b^Multiple regression (adjusted for age, gender and for all the variables in the model, Nagelkerke *R* ^2^ = 0.341; *χ* ^2^ = 121.731; *p* < 0.001)

**Table 6:** Ordinal regression analysis of predictors of EQ-5D self care domain

Patient characteristics	Univariate regression^a^		Multiple regression^b^	
	OR (95% CI)	*p*	OR (95% CI)	*p*
Years of education	1.99 (1.14–3.45)	0.015	1.92 (0.96–3.85)	0.067
Marital status	2.49 (1.17–5.29)	0.018	2.71 (1.09–6.71)	0.031
Duration of practice involvement >10 years	1.55 (0.71–3.38)	0.267		
Family physician visit frequency	0.41 (0.26–0.64)	<0.001	0.40 (0.24–0.67)	<0.001
Angina pectoris	0.73 (0.38–1.43)	0.361		
Myocardial infarction	0.46 (0.22–0.95)	0.037	0.43 (0.19–0.98)	0.045
Stent/coronary bypass	0.74 (0.32–1.71)	0.480		
Heart failure	0.22 (0.10–0.47)	<0.001	0.32 (0.14–0.74)	0.007
Stroke	0.20 (0.08–0.47)	<0.001	0.30 (0.10–0.87)	0.026
Peripheral artery disease	0.28 (0.14–0.56)	<0.001	0.45 (0.21–0.99)	0.046
Anxiety/depression	0.26 (0.13–0.51)	<0.001	0.34 (0.15–0.74)	0.007
Medication adherence (Morisky)	1.89 (0.28–11.45)	0.535		
Patient satisfaction (EUROPEP)	0.91 (0.54–1.55)	0.732		
Patient assessment of chronic illness care (PACIC)	0.87 (0.62–1.24)	0.443		

^a^Univariate regression (adjusted for age and gender)

^b^Multiple regression (adjusted for age, gender and for all the variables in the model, Nagelkerke *R* ^2^ = 0.394; *χ* ^2^ = 92.792; *p* < 0.001)

**Table 7:** Ordinal regression analysis of predictors of EQ-5D usual activities domain

Patient characteristics	Univariate regression^a^		Multiple regression^b^	
	OR (95% CI)	*p*	OR (95% CI)	*p*
Years of education	1.93 (1.45–2.59)	<0.001	1.73 (1.26–2.37)	0.001
Marital status	0.87 (0.53–1.41)	0.573		
Duration of practice involvement >10 years	1.03 (0.66–1.62)	0.883		
Family physician visit frequency	0.53 (0.40–0.71)	<0.001	0.58 (0.43–0.80)	0.001
Angina pectoris	0.76 (0.51–1.11)	0.156		
Myocardial infarction	0.74 (0.50–1.10)	0.141		
Stent/coronary bypass	0.64 (0.35–1.16)	0.142		
Heart failure	0.43 (0.28–0.66)	<0.001	0.61 (0.39–0.96)	0.034
Stroke	0.82 (0.39–1.72)	0.600		
Peripheral artery disease	0.37 (0.24–0.58)	<0.001	0.53 (0.33–0.84)	0.008
Anxiety/depression	0.22 (0.14–0.34)	<0.001	0.27 (0.17–0.44)	<0.001
Medication adherence (Morisky)	1.08 (0.35–3.37)	0.893		
Patient satisfaction (EUROPEP)	1.07 (0.77–1.49)	0.702		
Patient assessment of chronic illness care (PACIC)	1.02 (0.82–1.26)	0.867		

^a^Univariate regression (adjusted for age and gender)

^b^Multiple regression (adjusted for age, gender and for all the variables in the model, Nagelkerke *R* ^2^ = 0.295; *χ* ^2^ = 112.876; *p* < 0.001)

**Table 8:** Ordinal regression analysis of predictors of EQ-5D pain/discomfort domain

Patient characteristics	Univariate regression^a^		Multiple regression^b^	
	OR (95% CI)	*p*	OR (95% CI)	*p*
Years of education	1.96 (1.44–2.67)	<0.001	1.75 (1.26–2.44)	0.001
Marital status	1.87 (1.09–3.26)	0.023	2.08 (1.17–3.70)	0.013
Duration of practice involvement >10 years	0.85 (0.53–1.36)	0.487		
Family physician visit frequency	0.69 (0.51–0.93)	0.015	0.79 (0.57–1.10)	0.157
Angina pectoris	0.75 (0.49–1.13)	0.169		
Myocardial infarction	0.94 (0.61–1.43)	0.757		
Stent/coronary bypass	0.64 (0.34–1.21)	0.171		
Heart failure	0.33 (0.20–0.53)	<0.001	0.48 (0.29–0.81)	0.005
Stroke	0.73 (0.33–1.64)	0.451		
Peripheral artery disease	0.27 (0.16–0.44)	<0.001	0.37 (0.22–0.63)	<0.001
Anxiety/depression	0.12 (0.07–0.21)	<0.001	0.14 (0.08–0.25)	<0.001
Medication adherence (Morisky)	2.08 (0.62–6.97)	0.234		
Patient satisfaction (EUROPEP)	1.29 (0.91–1.84)	0.154		
Patient assessment of chronic illness care (PACIC)	1.15 (0.92–1.44)	0.231		

^a^Univariate regression (adjusted for age and gender)

^b^Multiple regression (adjusted for age, gender and for all the variables in the model, Nagelkerke *R* ^2^ = 0.388; *χ* ^2^ = 154.096; *p* < 0.001)

**Table 9:** Ordinal regression analysis of predictors of EQ-5D anxiety/depression domain

Patient characteristics	Univariate regression^a^		Multiple regression^b^	
	OR (95% CI)	*p*	OR (95% CI)	*p*
Years of education	1.54 (1.14–2.07)	0.005	1.44 (1.04–1.98)	0.027
Marital status	0.91 (0.55–1.49)	0.705		
Duration of practice involvement >10 years	0.98 (0.62–1.55)	0.940		
Family physician visit frequency	0.72 (0.54–0.95)	0.020	0.73 (0.54–0.98)	0.038
Angina pectoris	0.77 (0.52–1.15)	0.207		
Myocardial infarction	0.89 (0.60–1.34)	0.592		
Stent/coronary bypass	0.33 (0.18–0.58)	<0.001	0.38 (0.21–0.70)	0.002
Heart failure	0.58 (0.38–0.88)	0.012	0.74 (0.47–1.16)	0.185
Stroke	0.60 (0.28–1.26)	0.175		
Peripheral artery disease	0.42 (0.28–0.66)	<0.001	0.50 (0.32–0.79)	0.002
Anxiety/depression				
Medication adherence (Morisky)	1.22 (0.39–3.81)	0.739		
Patient satisfaction (EUROPEP)	1.62 (1.16–2.25)	0.004	1.71 (1.21–2.42)	0.003
Patient assessment of chronic illness care (PACIC)	1.12 (0.90–1.40)	0.294		

^a^Univariate regression (adjusted for age and gender)

^b^Multiple regression (adjusted for age, gender and for all the variables in the model, Nagelkerke *R* ^2^ = 0.149; *χ* ^2^ = 59.912; *p* < 0.001)

**Table 10:** Regression analysis of comorbidities as predictors of EQ-VAS score and EQ-5D

Patient characteristics	EQ-VAS^a^			EQ-5D^b^	
	B (95% CI)	SE	*p*	OR (95% CI)	*p*
Angina pectoris	0.16 (−3.47, 3.80)	1.85	0.931	0.78 (0.55–1.10)	0.151
Myocardial infarction	0.86 (−2.79, 4.50)	1.85	0.645	0.75 (0.53–1.05)	0.098
Stent/coronary bypass	2.62 (−2.88, 8.11)	2.79	0.350	1.17 (0.69–1.67)	0.562
Heart failure	−7.49 (−11.40, -3.57)	2.00	<0.001	0.39 (0.27–0.56)	<0.001
Stroke	2.11 (−5.00, 9,23)	3.62	0.559	0.83 (0.44–1.58)	0.575
Peripheral artery disease	−2.32 (−6.35, 1.72)	2.05	0.260	0.33 (0.22–0.48)	<0.001
Anxiety/depression	−11.71 (−15.50, -7.91)	1.93	<0.001		

^a^Multiple linear regression (adjusted for age, gender and for all the comorbidities in the model, *R* ^2^ = 0.217; F = 11.818; *p* < 0.001)

^b^Multiple ordinal logistic regression (adjusted for age, gender and for all the comorbidities in the model, Nagelkerke *R* ^2^ = 0.239; *χ* ^2^ = 114.427; *p* < 0.001)

## Abbreviations

CHD: Coronary heart disease
CVD: Cardiovascular diseases
EPA Cardio: European practice assessment of cardiovascular risk management
EQ-5D: European quality of life (EuroQoL) - 5 dimensions
EQ-VAS: EQ visual analogue scale
EUROPEP: European Project on Patient Evaluation of General Practice Care
HF: Heart failure
HRQoL: Health-related quality of life
ICD-10: International Statistical Classification of Diseases and Related Health Problems, 10th Revision
PACIC: Patient assessment of chronic illness care
PAD: Peripheral artery disease

## References

[1] Leal J, Luengo-Fernandez R, Gray A, Petersen S, Rayner M (2006). Economic burden of cardiovascular diseases in the enlarged European Union. Eur Heart J.

[2] Global Burden of Disease Study 2013 Collaborators (2015). Global, regional, and national incidence, prevalence, and years lived with disability for 301 acute and chronic diseases and injuries in 188 countries, 1990–2013: a systematic analysis for the Global Burden of Disease Study 2013. Lancet.

[3] Roth AG, Huffman MD, Moran AE, Feigin V, Mensah GA, Naghavi M (2015). Global burden of cardiovascular disease. Global and regional patterns in cardiovascular mortality from 1990 to 2013. Circulation.

[4] Kesteloot H, Sans S, Kromhout D (2006). Dynamics of cardiovascular and all-cause mortality in Western and Eastern Europe between 1970 and 2000. Eur Heart J.

[5] Mitchell PM, Al-Janabi H, Richardson J, Iezzi A, Coast J (2015). The relative impacts of disease on health status and capability wellbeing: a multi-country study. PLoS One.

[6] Muhammad I, He HG, Kowitlawakul Y, Wang W (2014). Narrative review of health-related quality of life and its predictors among patients with coronary heart disease. Int J Nurs Pract.

[7] Dickens C, Cherrington A, McGowan L (2012). Depression and health-related quality of life in people with coronary heart disease: a systematic review. Eur J Cardiovasc Nurs.

[8] Wilson IB, Cleary PD (1995). Linking clinical variables with health related quality of life. JAMA.

[9] Rumsfeld JS (2002). Health status and clinical practice: when will they meet?. Circulation.

[10] Kramer L, Hirsch O, Schlöβler K, Träger S, Baum E, Donner-Banzhoff N (2012). Associations between demographic, disease related, and treatment pathway related variables and health related quality of life in primary care patients with coronary heart disease. Health and Qual Life Outcomes.

[11] Rector TS, Anand IS, Cohn JN (2006). Relationships between clinical assessments and patients’ perceptions of the effects of heart failure on their quality of life. J Card Fail.

[12] Stauber S, Schmid JP, Saner H, Znoj H, Saner G, Grolimund J, Von Känel R (2013). Health-related quality of life is associated with positive affect in patients with coronary heart disease entering cardiac rehabilitation. J Clin Psychol Med Settings.

[13] Mommersteeg P, Denollet J, Spertus J, Pedersen S (2009). Health status as a risk factor in cardiovascular disease: a systematic review of current evidence. Am Heart J.

[14] Comin-Colet J, Lainscak M, Dickstein K, Filippatos GS, Johnson P, Lüscher TF (2013). The effect of intravenous ferric carboxymaltose on health-related quality of life in patients with chronic heart failure and iron deficiency: a subanalysis of the FAIR-HF study. Eur Heart J.

[15] Westin L, Nilstun T, Carlsson R, Erhardt L (2005). Patients with ischemic heart disease: quality of life predicts long-term mortality. Scand Cardiovasc J.

[16] Höfer S, Benzer W, Oldridge N (2014). Change in health-related quality of life in patients with coronary artery disease predicts 4-year mortality. Int J Cardiol.

[17] Coons SJ, Rao S, Keininger DL, Hays RD (2000). A comparative review of generic quality-of-life instruments. Pharmacoeconomics.

[18] Wensing M, Ludt S, Campbell S, van Lieshout J, Volbracht E, Grol R (2009). European practice assessment of cardiovascular risk management (EPA cardio): protocol of an international observational study in primary care. Implement Sci.

[19] Campbell SM, Ludt S, Van Lieshout J, Boffin N, Wensing M, Petek D (2008). Quality indicators for the prevention and management of cardiovascular disease in primary care in nine European countries. Eur J Cardiovasc Prev Rehabil.

[20] Greiner W, Weijnen T, Nieuwenhuizen M, Oppe S, Badia X (2003). A single European currency for EQ-5D health states. Results from a six-country study. Eur J Health Econ.

[21] Rabin R, de Charro F (2001). EQ-5D: a measure of health status from the EuroQol Group. Ann Med.

[22] Prevolnik Rupel V, Ogorevc M (2012). The EQ-5D health states value set for Slovenia. Zdrav Varst.

[23] Dyer MT, Goldsmith KA, Sharples LS, Buxton MJ (2010). A review of health utilities using the EQ-5D in studies of cardiovascular disease. Health Qual Life Outcomes.

[24] Morisky DE, Ang A, Krousel-Wood M, Ward HJ (2008). Predictive validity of a medication adherence measure in an outpatient setting. J Clin Hypertens (Greenwich).

[25] Schmittdiel J, Mosen DM, Glasgow RE, Hibbard J, Remmers C, Bellows J (2008). Patient assessment of chronic illness care (PACIC) and improved patient-centered outcomes for chronic conditions. J Gen Intern Med.

[26] Gugiu C, Coryn CL, Applegate B (2010). Structure and measurement properties of the patient assessment of chronic illness care instrument. J Eval Clin Pract.

[27] Kersnik J (2000). An evaluation of patient satisfaction with family practice care in Slovenia. Int J Qual Health Care.

[28] Grol R, Wensing M, Mainz J, Jung HP, Ferreira P, Hearnshaw H (2000). Patients in Europe evaluate general practice care: an international comparison. Br J Gen Pract.

[29] Vierron E, Giraudeau B (2009). Design effect in multicenter studies: gain or loss of power?. BMC Med Res Methodol.

[30] Donner A, Birkett N, Buck C (1981). Randomization by cluster. Sample size requirements and analysis. Am J Epidemiol.

[31] Smeeth L, Ng ES (2002). Intraclass correlation coefficients for cluster randomized trials in primary care: data from the MRC trial of the assessment and management of older people in the community. Control Clin Trials.

[32] Faul F, Erdfelder E, Buchner A, Lang AG (2009). Statistical power analyses using G*power 3.1: tests for correlation and regression analyses. Behav Res Meth.

[33] Cohen J (1988). Statistical power analysis for the behavioral sciences.

[34] Hsieh FY (1989). Sample size tables for logistic regression. Stat Med.

[35] Rosenthal JA (1996). Qualitative descriptors of strength of association and effect size. J Soc Serv Res.

[36] Hsieh FY, Lavori PW, Cohen HJ, Feussner JR (2003). An overview of variance inflation factors for sample-size calculation. Eval Health Prof.

[37] Alava MH, Wailoo AJ, Ara R (2012). Tails from the peak district: adjusted limited dependent variable mixture models of EQ-5D questionnaire health state utility values. Value Health.

[38] Ose D, Rochon J, Campbell SM, Wensing M, van Lieshout J, Uhlmann L (2012). Secondary prevention in patients with coronary heart diseases: what factors are associated with health status in usual primary care?. PLoS One.

[39] Wang L, Wu YQ, Tang X, Li N, He L, Cao Y (2015). Profile and correlates of health-related quality of life in Chinese patients with coronary heart disease. Chin Med J (Engl).

[40] Xie J, Wu EQ, Zheng ZJ, Sullivan PW, Zhan L, Labarthe DR (2008). Patient-reported health status in coronary heart disease in the United States: age, sex, racial, and ethnic differences. Circulation.

[41] Burström K, Johannesson M, Diderichsen F (2001). Swedish population health-related quality of life results using the EQ-5D. Qual Life Res.

[42] Bell RJ, Rivera-Woll L, Davison SL, Topliss DJ, Donath S, Davis SR (2007). Wellbeing, health-related quality of life and cardiovascular disease risk profile in women with subclinical thyroid disease – A community-based study. Clin Endocrinol (Oxf).

[43] Calvert MJ, Freemantle N, Cleland JG (2005). The impact of chronic heart failure on health-related quality of life data acquired in the baseline phase of the CARE-HF study. Eur J Heart Fail.

[44] Juenger J, Schellberg D, Kraemer S, Haunstetter A, Zugck C, Herzog W (2002). Health related quality of life in patients with congestive heart failure: comparison with other chronic diseases and relation to functional variables. Heart.

[45] Hobbs FD, Kenkre JE, Roalfe AK, Davis RC, Hare R, Davies MK (2002). Impact of heart failure and left ventricular systolic dysfunction on quality of life: a cross-sectional study comparing common chronic cardiac and medical disorders and a representative adult population. Eur Heart J.

[46] Hertzer NR, Beven EG, Young JR, O’Hara PJ, Ruschhaupt WF, Graor RA (1984). Coronary artery disease in peripheral vascular patients. A classification of 1000 coronary angiograms and results of surgical management. Ann Surg.

[47] de Graaff JC, Ubbink DT, Kools EI, Chamuleau SA, Jacobs MJ (2002). The impact of peripheral and coronary artery disease on health-related quality of life. Ann Vasc Surg.

[48] Long J, Modrall JG, Parker BJ, Swann A, Welborn MB, Anthony T (2004). Correlation between ankle-brachial index, symptoms, and health-related quality of life in patients with peripheral vascular disease. J Vasc Surg.

[49] Levine GN, Bates ER, Blankenship JC, Bailey SR, Bittl JA, Cercek B (2011). 2011. ACCF/AHA/SCAI guideline for percutaneous coronary intervention: executive summary: a report of the American college of cardiology foundation/American heart association task force on practice guidelines and the society for cardiovascular angiography and interventions. Circulation.

[50] Rumsfeld JS, Magid DJ, O’Brien M, McCarthy M, MaWhinney S, Scd, Shroyer AL (2001). Changes in health-related quality of life following coronary artery bypass graft surgery. Ann Thorac Surg.

[51] Suominen-Taipale AL, Koskinen S, Martelin T, Holmen J, Johnsen R (2004). Differences in older adults’ use of primary and specialist care services in two Nordic countries. Eur J Public Health.

[52] Redondo-Sendino A, Guallar-Castillón P, Banegas JR, Rodríguez-Artalejo F (2006). Gender differences in the utilization of health-care services among the older adult population of Spain. BMC Public Health.

[53] Vegda K, Nie JX, Wang L, Tracy CS, Moineddin R, Upshur RE (2009). Trends in health services utilization, medication use, and health conditions among older adults: a 2-year retrospective chart review in a primary care practice. BMC Health Serv Res.

[54] Kersnik J, Svab I, Vegnuti M (2001). Frequent attenders in general practice: quality of life, patient satisfaction, use of medical services and GP characteristics. Scan J Prim Health Care.

[55] Skodova Z, van Dijk JP, Nagyova I, Rosenberger J, Ondusova D, Studencan M (2010). Psychosocial factors of coronary heart disease and quality of life among Roma coronary patients: a study matched by socioeconomic position. Int J Public Health.

[56] Street RL, Makoul G, Arora NK, Epstein RM (2009). How does communication heal? Pathways linking clinician-patient communication to health outcomes. Patient Educ Couns.

[57] Billups SJ, Malone DC, Carter BL (2000). The relationship between drug therapy noncompliance and patient characteristics, health-related quality of life and health care costs. Pharmacotherapy.

[58] Holt EW, Muntner P, Joyce CJ, Webber L, Krousel-Wood MA (2010). Health-related quality of life and antihypertensive medication adherence among older adults. Age Ageing.

[59] Turpin RS, Simmons JB, Lew JF, Alexander CM, Dupee MA, Kavanagh P (2004). Improving treatment regimen adherence in coronary heart disease by targeting patient types. Dis Manag Health Out.

